# Absolute quantification of redox state of cytochrome c oxidase and hemoglobin in human tissue using continuous-wave broadband near-infrared spectroscopy

**DOI:** 10.21203/rs.3.rs-7760901/v1

**Published:** 2025-10-05

**Authors:** Fatemeh Tavakoli, Xinlong Wang, Hanli Liu

**Affiliations:** University of Texas at Arlington; University of Texas at Arlington; University of Texas at Arlington

**Keywords:** Broadband near-infrared spectroscopy, continuous-wave, absolute quantification, cytochrome c oxidase, oxyhemoglobin, deoxyhemoglobin, tissue components

## Abstract

Using the diffusion approximation, we developed a continuous-wave (CW) broadband near-infrared spectroscopy (bb-NIRS) system to noninvasively quantify the absolute concentrations of redox state of cytochrome c oxidase [CCO] and other major human tissue chromophores. The algorithm leverages characteristic spectral features obtained from the first and second derivative domains of wavelength-dependent extinction coefficients. The validation of the method was performed through computational simulations to evaluate estimation accuracy, followed by in vivo measurements on the forearms of 20 participants and the foreheads of 18 participants. The recovered values showed strong agreement with established physiological parameters within maximum of 10% deviation, prior literature, and concurrent frequency-domain near-infrared spectroscopy (FD-NIRS) measurements. These findings demonstrate the feasibility of a CW-based approach for accurate and noninvasive absolute quantification of human tissue components.

## 1. Introduction

Near-infrared spectroscopy (NIRS) is a noninvasive optical technique that uses near-infrared light to quantify the concentrations of oxygenated hemoglobin [HbO] and deoxygenated hemoglobin [HHb] by analyzing their wavelength-dependent extinction coefficients and the scattering properties of biological tissues [1, 2]. This method is widely used to evaluate hemoglobin oxygenation levels and tissue scattering properties in various human tissues [3–5]. Depending on the light modulation and detection approach, NIRS can be broadly categorized into three major modalities, including time-domain (TD), frequency-domain (FD), and continuous-wave (CW) systems.

TD-NIRS system determines tissue absorption and scattering properties by analyzing the histogram of photon time-of-flight, made possible by high-speed photon emitters and detectors. Using this technique, Montcel et al. quantified depth-resolved cerebral hemodynamics [6], and Brady et al. explored cerebrovascular autoregulation in acute brain injury through its pressure-passive dynamics [7]. A FD-NIRS system utilizes sinusoidal amplitude modulation, typically in the order of 100 MHz, of laser sources. By analyzing the phase delay and amplitude attenuation of the diffused light, the FD-NIRS system provides absolute measurements of tissue absorption and scattering. For instance, Fantini et al. employed FD-NIRS to estimate hemoglobin concentrations and scattering coefficients in the foreheads of both young and older adults [8, 9]. While both time- and FD-NIRS systems offer high accuracy in quantifying optical properties, they require complex and expensive instrumentation. The third modality, CW-NIRS, operates with non-modulated light and uses slower detectors such as photodiodes or spectrometers [10, 11]. CW-NIRS system allows for measuring relative changes in chromophore concentrations from attenuated optical density based on the modified Beer–Lambert Law. Although CW-NIRS cannot independently separate absorption and scattering effects due to the use of non-modulated light and the limited temporal information from source and detector, it remain more cost-effective and compact compared to TD-NIRS and FD-NIRS instruments [12]. Furthermore, CW-NIRS can be configured for broadband NIRS (bb-NIRS), enabling simultaneous measurements across a wide spectral range. This broadband capability enhances quantification by exploiting the wavelength-dependent absorption features of chromophores [13, 14].

Most NIRS quantification algorithms rely on the simplifying assumption that only [HbO] and [HHb], sometimes with water, contribute to absorption, which usually result in undesirable errors [15]. However, actual human tissue contains additional chromophores with physiologically meaningful absorption features. One key contributor is cytochrome c oxidase [CCO], the terminal enzyme of the mitochondrial electron transport chain, which accounts for roughly 95% of oxygen metabolism and drives adenosine triphosphate (ATP) synthesis [13, 16, 17]. The redox state of [CCO], defined as oxidized [CCO] minus reduced [CCO], directly reflects mitochondrial oxygen utilization and energy metabolism. For brevity, we denote the redox state of CCO as [CCO] throughout the paper. Alterations in [CCO] concentration are directly linked to cellular oxygenation, energy supply, and tissue health [18–20]. Given its high absorption in the near-infrared range, [CCO] is detectable by NIRS, particularly with bb-NIRS implementations [14, 21].

Building on the Modified Beer–Lambert Law, Matcher et al. first demonstrated the use of multi-wavelength NIRS to quantify [CCO] changes in 1995[22]. Subsequent work expanded on this foundation, where Ghosh et al. developed a hybrid probe that incorporated the wavelength dependency of the differential pathlength factor (DPF) to improve estimates of [CCO] changes during cerebral activation[18]. Using a similar broadband approach, Wang et al. later investigated [CCO] dynamics in response to photobiomodulation and thermal stimulation [23–25]. However, these studies were limited to relative quantification, which is highly sensitive to motion artifacts and instrumental noise [26]. To enable absolute quantification, Matcher et al. proposed using water spectral features derived from extinction spectra[27]. This strategy was later refined by Lawrence et al., who applied CW bb-NIRS to estimate [HbO], [HHb], water content, and scattering coefficients based on their characteristic derivative spectra [28]. Notably, [CCO] was excluded from these studies, and the fitting algorithms lacked computational validation to support estimation reliability.

In this study, for the first time, we present a derivation-based CW-NIRS system for absolute quantification of redox state of [CCO] along with other tissue components, including [HbO], [HHb], water%, fat%, and scattering coefficient factors (scatterer size and power). The algorithm was developed within the diffusion theory framework, incorporating mathematical derivations and simplifications for efficient fitting. Broadband CW-NIRS spectra were acquired from human tissue and analyzed within the diffusion approximation framework, where first- and second-derivative spectra were fitted to simplified photon flux equations. A multi-step ant-colony optimization algorithm was implemented to recover chromophore concentrations and scattering properties efficiently. The method was validated through simulations to evaluate estimation accuracy and subsequently tested in vivo on human foreheads and forearms. Results demonstrated strong consistency with concurrent FD-NIRS measurements, prior studies, and well-established physiological expectations [9, 13, 29, 30].

## 2. Materials and methods

### 2.1 Mathematical modeling of photon propagation

Photon propagation in turbid biological tissue under continuous-wave illumination can be approximated using the diffusion equation. The photon flux detected at a source–detector separation ρ is expressed as [31]:

1 $$R\left(\rho ,z_{0}\right)=\frac{1}{4\pi }\left[z_{0}\left(\mu_{eff}+\frac{1}{r_{1}}\right)\frac{e^{-\mu_{eff}r_{1}}}{r_{1}^{2}}+\left(z_{0}+4AD\right)\left(\mu_{eff}+\frac{1}{r_{2}}\right)\frac{e^{-\mu_{eff}r_{1}}}{r_{2}^{2}}\right]$$

where $\mu_{\text{eff}}=\sqrt{\mu_{a}/D}$ is the effective attenuation coefficient, $D={\left[3\left(\mu_{a}+\mu_{s}^{\prime }\right)\right]}^{-1}$ is the diffusion coefficient (cm), $\mu_{a}({cm}^{-1})$ is the absorption coefficient, $\mu_{s}^{\prime }\left({cm}^{-1}\right)$ is the reduced scattering coefficient. The terms $r_{1}=\sqrt{z_{0}^{2}+\rho^{2}},\;r_{2}=\sqrt{{\left(z_{0}+4AD\right)}^{2}+\rho^{2}}$ represent the distances from the detector to the real isotropic source and the extrapolated image source, respectively. These distances are defined based on source detector separation ρ (cm) and the depth of the real isotropic source below the tissue boundary as $z_{0}=3D$. The constant A accounts for the mismatch of refractive indices at the tissue–air interface [32].

To account for the wavelength dependence of scattering, $\mu_{s}^{\prime }$ is modeled by a power-law relationship based on Mie theory [33, 34] as:

2 $$\mu_{s}^{\prime }(\lambda )=m{\left(\frac{\lambda }{750}\right)}^{-n}$$

where λ (nm) is the wavelength, m (cm^−1^) is the scattering amplitude related to the density and size of scatterers, and n (dimensionless) is the scattering power that describes the wavelength dependence of scattering [33–35]. In this study, spectra in the range of 650–980 nm were analyzed to capture absorption features of [HbO], [HHb], water, lipid, and [CCO].

For in vivo experiments, the source–detector separations were set to 1.5 cm on the forearm and 3.0 cm on the forehead. At these separations, the typical tissue optical properties were approximately $\mu_{a}\approx 0.1\;{cm}^{-1}$ and $\mu_{s}^{\prime }=10\;{cm}^{-1}$ [9]. Under these conditions, the magnitude of $\rho^{2}$ is on the order of 10^1^, whereas the scale of ${z_{0}}^{2}$ is on the order of 10^−2^, which is less than 1% of $\rho^{2}$. Therefore, ${z_{0}}^{2}$ can be neglected in Eq. (1). Furthermore, in the diffusion regime (i.e. $\mu_{a}\ll \mu_{s}^{\prime }$), the diffusion coefficient can be approximated as $D\approx {\left(3\mu_{s}^{\prime }\right)}^{-1}$.

By taking the natural logarithm of Eq. (1), the equation can be simplified into a logarithmic form as:

3 $$log\;(R)=log\;\left(1+\rho \sqrt{3\mu_{a}\mu_{s}^{\prime }}\right)-log\left(\mu_{s}^{\prime }\right)+C$$

where C is a wavelength-independent constant that can be eliminated by spectral differentiation.

**Figure 1:**
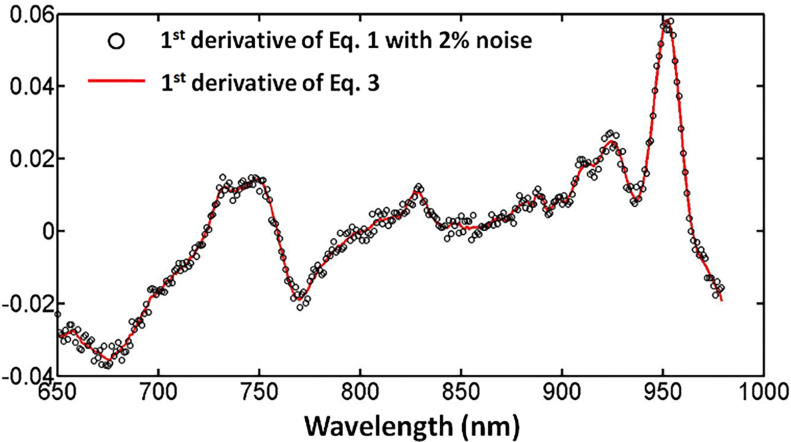
First-derivative spectra generated from the full photon diffusion approximation (using Eq. (1), black circles, with 2% noise) compared with the simplified logarithmic model (using Eq. (3), red line) over the wavelength range of 650 nm to 980 nm.

Figure 1 compares spectra generated using the full diffusion model in Eq. (1) (black circles, with added 2% uniform noise) [31] and the simplified logarithmic form in Eq. (3) (red curve). This comparison demonstrates that the algebraic simplification substantially reduces the mathematical complexity of the diffusion expression while preserving its spectral features and overall scale.

To further assess the feasibility and accuracy of this simplification, simulations were carried out in which Eq. (1) was used to generate simulated spectra resembling (measured data from human tissue), while Eq. (3) was applied to perform the fitting process. The relative error between fitted and simulated values was then calculated to quantify the effects introduced by the simplification.

### 2.2 Simulation and optimization algorithm

#### 2.2.1 Simulation setup and parameters

Based on values reported in the literature [9, 28–30], four representative tissue parameter sets were defined from prior NIRS literature to cover brain and skeletal muscle physiology (Table 1). Each set specifies [HbO], [HH], [CCO], water%, fat%, and the scattering size m and scattering power n describing the ${\mu^{\prime }}_{s}$, These parameter sets were treated as ground-truth references.

Simulations were performed across wavelengths ranging from 650 to 980 nm, generating multiple synthetic spectra using Eq. (1) described in section 2.1. To reduce fitting complexity, the upper and lower bounds of each fitting parameter were constrained within physiological ranges $[HbO]\in \left[0,\;80\right]\mu M$, $[HHb]\in \left[0,\;50\right]\mu M$, [CCO]∈[0,20]μM,water%∈[60%,95%], fat%∈[0,70%], $m\in [0,\;30]\;{cm}^{-1},\;n\in [0.7,\;1.5]$. For applying the algorithm to in vivo data, the scattering power n was limited to 0.9–1.1 for forearm tissue and 1.2–1.5 for forehead tissue, reflecting prior FD-NIRS measurements and minimizing variability. The hemoglobin concentrations were chosen following Fantini et al. [8, 9], while [CCO] was set to approximately 5–10% of the total hemoglobin concentration [13, 30]. The water% was fixed at 80%, and the fat% at 25% to approximate brain tissue composition. To assess robustness, random Gaussian noise was added to the simulated spectra at nine levels of 1%, 2%, 5%, 8%, 10%, 12%, 15%, 18%, and 20%. For each level, parameters were recovered by fitting Eq. (4) to the first-derivative (and where specified, second-derivative) spectra and compared to ground truth via percent relative error.

#### 2.2.2 Ant colony optimization algorithm

Parameter estimation was performed with an ant colony optimization algorithm (ACO) to minimize the cost function $\chi^{2}$ between modeled and simulated derivative spectra. ACO is a nature-inspired metaheuristic optimization algorithm originally proposed by Dorigo in 1992 [36–38]. The algorithm mimics the foraging behavior of ants where a population of ants explores candidate solutions sampled from pheromone-biased probability distributions. Pheromones are reinforced by low-cost solutions and evaporate otherwise, allowing the colony to gradually concentrate around promising regions of the search space while avoiding premature convergence.

We employed a continuous-domain variant with global-best reinforcement and pheromone evaporation applied at each iteration. Parameter bounds were enforced through clamping to physiologically defined ranges, as described in Section 2.2.1. Within this framework, ACO was used to recover the optimal combination of chromophore concentrations and scattering parameters that minimized the spectral mismatch between the simplified diffusion model (Eq. (3)) and the simulated CW spectra (Eq. (1)) in the first- and second-derivative domains.

#### 2.2.3 Fitting protocols and error metrics

To evaluate the performance of the proposed algorithm, three fitting protocols were tested. (i) minimizing $\chi_{1}^{2}$ on first-derivative spectra, (ii) minimizing $\chi_{2}^{2}$ on second-derivative spectra, and (iii) minimizing their sum $\chi_{1}^{2}+\chi_{2}^{2}$. The first derivative emphasizes spectral slopes and sensitivity to broad absorption features, while the second derivative enhances selectivity and suppresses baseline effects. Combining both error terms allows complementary information to be captured, thereby reducing inter-parameter cross-talk and improving robustness to noise. For each protocol and noise level, we report percent relative error between recovered parameters and ground truth. This design enables direct comparison of stability–accuracy trade-offs across derivative domains while holding forward and regularization assumptions fixed.

### 2.3 In vivo experiments

#### 2.3.1 Participants

Human participants were recruited from the local community of the University of Texas at Arlington (UTA). Eligibility was determined by investigator-led screening. Inclusion criteria were: (1) sex, (2) any ethnic background, and (3) age range 18–55 years. Exclusion criteria included: (1) psychiatric disorder diagnosis, (2) history of neurological condition, (3) severe brain injury, (4) violent behavior, (5) institutionalization or imprisonment, (6) current medication use, (7) diabetes diagnosis, (8) smoking, (9) excessive alcohol use, and (10) pregnancy.

A total of 20 participants (mean age: 33.8 ± 8.7 years) completed forearm experiments, and 18 participants (mean age: 31.5 ± 5.6 years) completed forehead experiments. Detailed demographic information and scattering properties measured by the FD-NIRS system are summarized in Table 2 for human forearm and forehead. All procedures were approved by the UTA Institutional Review Board (IRB), and written informed consent was obtained from each participant prior to participation.

#### 2.3.2 Instrumentation, data acquisition, and processing

**Figure 2:**
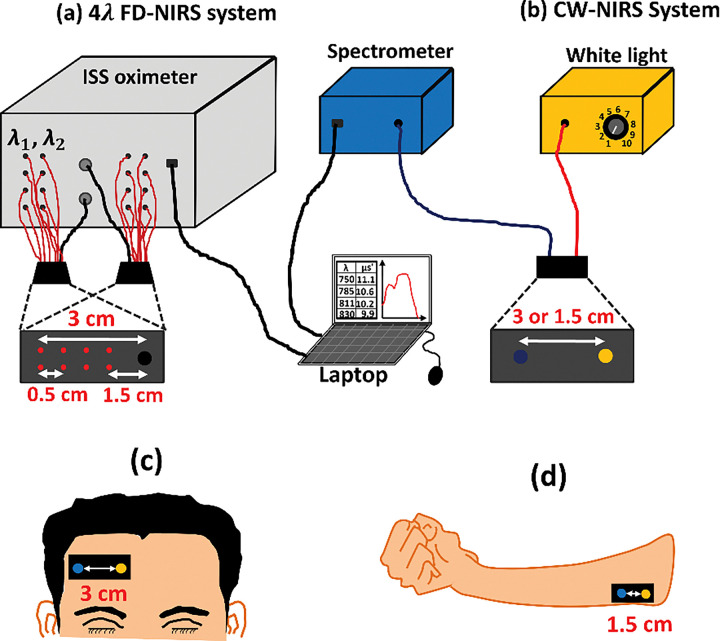
The experimental setup for FD-NIRS and CW-NIRS measurements. (a) FD-NIRS system (OxiplexTS, ISS Inc., Champaign, IL, USA) consists of two probes and four wavelengths (λ_1_ = 750 nm, λ_2_ = 785 nm, λ_3_ = 830 nm, and λ_4_ = 811 nm), with each probe detecting scattering and absorption properties at two wavelengths. Both probes share the same source detector geometry. (b) CW-NIRS measurement; the yellow box represents the broadband white-light source (Model 3900, Illumination Technologies, Inc., East Syracuse, NY, USA), and the blue box represents the spectrometer (i-trometer, B&W Tek Inc., Newark, DE, USA). Source–detector separations were 1.5 cm for forearm measurements and 3.0 cm for forehead measurements. (c) Measurement site for bb-NIRS on forehead with a source–detector separation of 3.0 cm, and (b) measurement sit for bb-NIRS on forearm with a source–detector separation of 1.5 cm.

A four-wavelength FD-NIRS system (OxiplexTS, ISS Inc., Champaign, IL, USA) was employed to measure tissue scattering coefficients. The system incorporated 16 laser diodes operating at $\lambda_{1}=750\;nm,\;\lambda_{2}=830\;nm,\;\lambda_{3}=785\;nm$, and $\lambda_{4}=811\;nm$, modulated at 110 MHz. Two optical probes were used, each equipped with four pairs of source fibers delivering light at two wavelengths and a single detector fiber for photon collection. Specifically, one probe measured at 750 and 830 nm, while the other measured at 785 and 811 nm. Source–detector separations of 1.5, 2.0, 2.5, and 3.0 cm were employed. Scattering coefficients were obtained at the four wavelengths under the semi-infinite boundary condition [8], and Mie theory was applied (Eq. 2) to determine m and n. The experimental setup is shown in Fig. 2(a).

Figure 2. The experimental setup for FD-NIRS and CW-NIRS measurements. (a) FD-NIRS system (OxiplexTS, ISS Inc., Champaign, IL, USA) consists of two probes and four wavelengths ($\lambda_{1}=750\;nm,\;\lambda_{2}=785\;nm,\;\lambda_{3}=830\;nm$, and $\lambda_{4}=811\;nm$), with each probe detecting scattering and absorption properties at two wavelengths. Both probes share the same source detector geometry. (b) CW-NIRS measurement; the yellow box represents the broadband white-light source (Model 3900, Illumination Technologies, Inc., East Syracuse, NY, USA), and the blue box represents the spectrometer (i-trometer, B&W Tek Inc., Newark, DE, USA). Source–detector separations were 1.5 cm for forearm measurements and 3.0 cm for forehead measurements. (c) Measurement site for bb-NIRS on forehead with a source–detector separation of 3.0 cm, and (b) measurement site for bb-NIRS on forearm with a source–detector separation of 1.5 cm.

One sample t-tests were performed on the fitted scattering power n, comparing values from all subjects against literature-reported values for both forearm and forehead tissues [29]. No significant differences were observed, comparing values from all subjects against literature-reported values for both forearm and forehead tissues. Therefore, the upper and lower bounds of factor n during the fitting were defined as [0.9 1.1] on human forearm and [1.2 1.4] on human forehead for the minimization of vulnerability to noise. Detailed explanation for the definition of narrow fitting range for factor n is provided in discussion section.

After the FD-NIRS measurement, from the same location on human tissue, a bb-NIRS system was used to obtain a continuous-wave spectrum. The bb-NIRS system consisted of a broadband white-light source (Model 3900, Illumination Technologies, Inc., East Syracuse, NY, USA) and a back-thinned CCD spectrometer (i-trometer, B&W Tek Inc., Newark, DE, USA). Photons were delivered to the tissue through an optical fiber bundle, and diffusely reflected light was collected at a source–detector separation of 3 cm on the forehead (1.5 cm for forearm) and transmitted to the spectrometer. To ensure consistency, both bb-NIRS and FD-NIRS probes had identical tissue-contact areas (5.0 × 2.0 cm^2^). Data were stored on a laptop and later used for simultaneous fitting of [HbO], [HHb], [CCO], water%, fat%, and scattering properties.

Raw spectra measured from human tissue were corrected by subtracting dark noise and calibrating against a reflectance standard to remove spectral artifacts [11, 38, 39]. The calibrated spectrum was computed as:

4 $$R_{1}(\lambda )=\frac{R_{0}(\lambda )\;-\;\text{dark}\;\text{noise}}{R_{r}(\lambda )\;-\;\text{dark}\;\text{noise}}$$

where $R_{0}(\lambda )$ is the raw spectrum measured from human tissue, $R_{r}(\lambda )$ is the reference spectrum from the reflectance standard, and $R_{1}(\lambda )$ is the calibrated spectrum used in the fitting process.

Figures 2(b) and 2(c) illustrates the specific measurement locations for the bb-NIRS system on human forearms and foreheads. For forehead experiments, the probe was placed on the right side near the hairline, consistent with previous studies [9, 23]. For forearm experiments, the flexor digitorum muscle was identified by asking participants to contract and relax their fists, and the measurement site was marked to ensure both systems probed the identical location [40].

Finaly, the comparison of relative errors from the simulation process showed that [HbO] was recovered with the highest accuracy using the first derivative fitting protocol. In contrast, the most accurate [HHb], [CCO], water%, fat%, and scattering parameters (m and n) were obtained using the combined-domain fitting protocol, which minimizes the total misfit $\chi_{1}^{2}+\chi_{2}^{2}$ (where $\chi_{1}^{2}$ and $\chi_{2}^{2}$ correspond to the goodness of fit in the first- and second-derivative domains, respectively). Therefore, during the process of parameter estimation (i.e., chromophore concentrations and scattering factors), we first applied the first derivative fitting to obtain the final [HbO]. The recovered values of the other parameters from this stage were then used as the initial guess of the second fitting. This two-step strategy leverages the robustness of the first derivative for [HbO] while enhancing the stability and accuracy of the remaining parameters through combined-domain fitting.

## 3. Results

### 3.1 Rectification of scattering power using FD-NIRS measurements

The FD-NIRS system was first used to quantify scattering properties of human tissue and compare them with values reported in the literature. Scattering coefficients were obtained at 750 nm, 785 nm, 811 nm, and 830 nm for both human forearm and forehead measurements. These coefficients were then fitted with Eq. (2) to calculate the scattering power factor based n on Mie Theory. The measured scattering coefficients and fitted n values for all participants are provided in Table 2.

**Figure 3:**
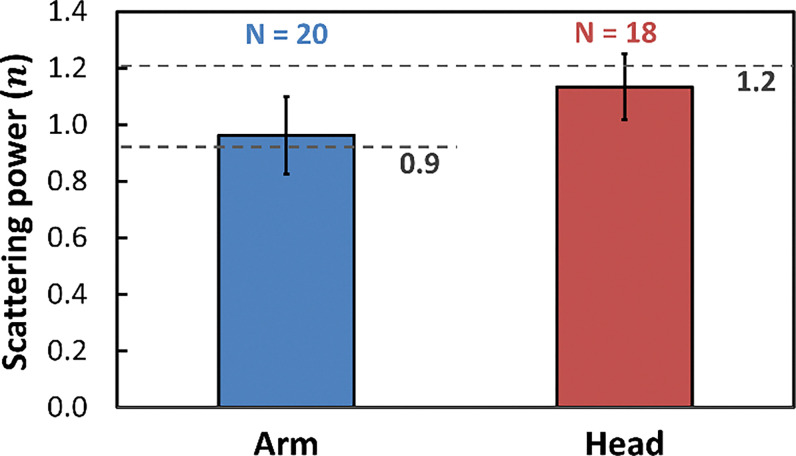
Comparison of scattering power factor (*n*) measured in human participants with literature-reported reference values (0.9 for human forearm and 1.2 for human forehead). Light gray bars represent forearm results (N = 20), and dark gray bars represent forehead results (N = 18). Error bars denote the standard deviation of mean. One-sample t-tests confirmed no significant differences between measured values and literature values.

Previous studies reported scattering power values of approximately n=0.9 for forearm tissue and n=1.2 for forehead tissue. Figure 3 compares the fitted n obtained from participants in this study with these literature values. One-sample t-tests were performed to compare the scattering power n measured in this study with literature-reported values. Specifically, n values from forearm measurements (N = 20) were tested against the reference value of 0.9, and n values from forehead measurements (N = 18) were tested against the reference value of 1.2. As shown in Fig. 3, no significant differences were observed between the measured results and the corresponding literature values. The sampling mean and standard deviation of n were calculated as 0.9 ± 0.1 for forearms and 1.1 ± 0.1 for foreheads. The limited inter-subject variability suggests that constraining the initial guess of n to the literature-reported values, within a narrow fitting range, would not bias subsequent parameter estimation.

### 3.2 Simulations

**Figure 4:**
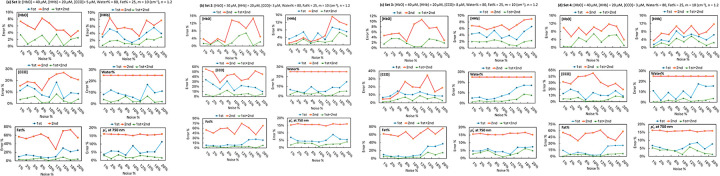
Relative error of the recovered parameters versus measurement noise level from 2% to 20% for four ground-truth parameter sets from Table 1: (a) Set 1, (b) Set 2, (c) Set 3, and (d) Set 4. Within each subfigure, six panels report the error for [HbO], [HHb], [CCO], water volume fraction (Water%), fat volume fraction (Fat%), and μ_s_ at 750 nm, respectively. Blue curves use first-derivative fitting only, green curves use second-derivative fitting only, and red curves use the combined first- and second-derivative fitting. These four subfigures collectively show how estimation accuracy depends on the underlying tissue composition (parameter sets 1–4 in Table 1) and on the choice of derivative feature set.

To quantify how noise impacts parameter recovery, we simulated spectra for the four ground-truth tissue compositions listed in Table 1 and added multiplicative noise from 2%–20% (2% steps). Each noisy dataset was fitted three ways: using the first derivative, the second derivative only, or the combined first- and second-derivative features. The resulting relative errors (defined as |true value – recovered value| / true value × 100%) for [HbO], [HHb], [CCO], water%, fat%, and $\mu_{s}^{\prime }$ at 750 nm are summarized in Fig. 4: subfigures (a)–(d) correspond to parameter Sets 1 to 4 in Table 1.

Across all four ground-truth sets, errors generally increased with noise. Using only the second derivative yielded the largest errors, particularly above ~ 10% noise, consistent with the higher noise amplification of second-order features. The first derivative was markedly more stable and, for [HbO] in particular, maintained low error across most noise levels. The combined first + second approach delivered the most consistent performance overall: it reduced errors for [HHb], [CCO], water, fat, and μs’ at 750 nm across the full noise range and remained comparatively robust even at 15–20% noise.

Among chromophores, [CCO] exhibited higher errors than [HbO] and [HHb] at a given noise level, reflecting its lower concentration and weaker spectral contrast. Nevertheless, for noise ≤ 10% its error typically remained around ~ 10%, indicating feasible quantification under realistic SNR. Because the recovered [HbO] from the first-derivative fit was used as input to the second-derivative fit, the blue curve (first-derivative only) is absent in the second-derivative panels for [HbO]. This design avoids redundancy and ensures consistency across fitting protocols. Note that the first derivative consistently provided the most reliable fit for [HbO] in our initial simulations. Therefore, the [HbO] value recovered from the first-derivative fit was fixed and used as input in the second-derivative fitting stage. As a result, the blue curve (first-derivative only) does not appear in the [HbO] panels for the second-derivative results. This ensures consistency across protocols and avoids redundancy while highlighting the superior performance of the first derivative for [HbO] recovery. Taken together, these results show that (i) first-derivative fitting is particularly reliable for [HbO], while (ii) the combined derivative strategy provides better overall stability for the remaining parameters, especially as noise increases, by leveraging complementary sensitivity in the two derivative orders.

### 3.3 In vivo measurements

To verify the robustness of the proposed method beyond simulations, we applied the same fitting framework to in vivo measurements of human tissue. The initial guesses and parameter bounds were identical to those used in simulations, except for the scattering power factor n, which was constrained to [0.9, 1.1] for forearm data and [1.2, 1.4] for forehead data.

**Figure 5:**
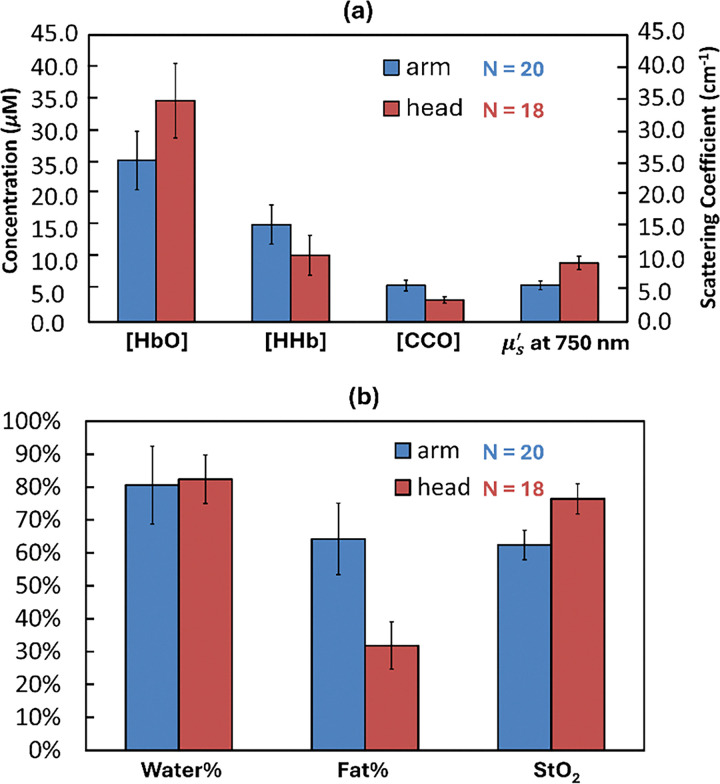
In vivo results in forearm (N = 20) and forehead (N = 18). (a) Mean [HbO], [HHb], and [CCO], together with at 750 nm. (b) Mean tissue water volume fraction (Water%), fat volume fraction (Fat%), and oxygen saturation (StO_2_). Bars show group means and error bars denote standard deviation of the mean across subjects. Colors correspond to cohorts: blue = forearm, orange = forehead.

Results from 20 forearms and 18 foreheads are summarized in Fig. 5. The top panel (Fig. 5(a)) reports recovered [HbO], [HHb], [CCO], and $\mu_{s}^{\prime }$ at 750 nm. On average, [HbO] was 25.3 *μ*M in forearms and 34.7 *μ*M in foreheads, [HHb] averaged 15.3 *μ*M in forearms and 10.5 *μ*M in foreheads. The mean [CCO] was 5.7 *μ*M in forearms and 3.4 *μ*M in foreheads, corresponding to approximately 7–14% of the total hemoglobin concentration, a ratio consistent with known physiological ranges [13, 15, 21, 30]. Additionally, the fitted $\mu_{s}^{\prime }$ at 750 nm averaged 5.6 cm^−1^ in forearms and 9.3 cm^−1^ in foreheads. The fitted $\mu_{s}^{\prime }$ at 750 nm averaged 5.6 cm^−1^ in forearm and 9.3 cm^−1^ in forehead. The bottom panel (Fig. 5(b)) shows water %, fat %, and oxygen saturation (StO_2_). The average water% was 80.6% in forearms and 82.3% in foreheads, while the fat% was 64.2% in forearms and 31.8% in foreheads. StO_2_, calculated from hemoglobin concentrations, averaged 62.4% in forearms and 76.4% in foreheads, which are consistent with physiological expectations, as higher cerebral oxygenation is required in brain than in forearm to support continuous neuronal activity and metabolic demand. Finally, bb-NIRS–derived $\mu_{s}^{\prime }$ at 750 nm values were compared with concurrent DF-NIRS measurements (Table 2) using two-sample *t*-tests. No significant differences were observed, supporting the validity and robustness of the ACO-based fitting model for in vivo human data.

## 4. Discussion

We developed and validated a non-invasive bb-NIRS system capable of quantifying absolute chromophores concentrations, water% and fat%, and scattering properties in biological tissues by leveraging the spectral features of first and second derivatives of continuous diffused reflectance spectra. Computational simulations were initially employed to assess the stability and estimation accuracy of the fitting algorithm under different noise levels, followed by in vivo experiments on human forearms and foreheads. The results yielded physiologically consistent values for [HbO], [HHb], [CCO], water%, fat%, and $\mu_{s}^{\prime }$, underscoring the feasibility and potential of this optical system for tissue component quantification and, ultimately, functional imaging applications.

### 4.1 Noise-dependent fitting behavior

A persistent challenge in derivative-spectrum fitting is parameter identifiability in the presence of noise, especially for the scattering power n. To visualize how noise reshapes the objective, we computed the cost surface $\chi^{2}(m,n)$ on a grid ($m\in \left[0,30\;{cm}^{-1}\right],\;n\in [0,3]$) using synthetic data generated at known chromophore concentrations and scattering. The results are shown in Fig. S1 in Supplemental Document. For each noise level (2.0%, 10.0%, and 18.0%), the figure shows: (a) the 3D $\chi^{2}$ surface; (b) a 2D heatmap of $\chi^{2}$ across m and n grid; and (c–d) one-dimensional slices taken through the cost minimum, where panel (c) plots $\chi^{2}$ versus n evaluated at the m that minimizes $\chi^{2}$, and panel (d) plots $\chi^{2}$ versus m evaluated at the n that minimizes $\chi^{2}$. The minimum is marked by a red dot.

To quantify how “sharp” the minimum is along each slice, we report a **contrast** measure: the percentage change between the largest and smallest $\chi^{2}$ values along that slice (i.e., the percent rise from the minimum to the maximum within the slice). Large contrast implies a steep, well-defined basin (high identifiability); small contrast indicates a relatively flat basin in which many parameter values fit almost equally well (low identifiability).

At a lower noise of 2.0% (Fig. S1 panel (I)), the $\chi^{2}$ landscape forms a deep, single basin with the minimum close to the ground-truth region (approximately $\approx 10\;{cm}^{-1},\;n\approx 1.1-1.2$. The one-dimensional slices through this minimum are highly contrasted with 6,567.4% for $\chi^{2}$ as a function of n (evaluated at the best m that minimizes $\chi^{2}$) and 26,939% for $\chi^{2}$ as a function of m (evaluated at the best n that minimizes $\chi^{2}$). These large contrasts indicate steep curvature and strong identifiability in both directions, with m being especially robust. At moderate noise level of 10.0% (Fig. S1 panel (II)), the landscape broadens, most noticeably along the n direction. The slice contrast for n drops to 238.1%, whereas m remains comparatively well-defined at 969.1%. Thus, while m can still be recovered reliably, n becomes substantially more sensitive to noise and more prone to cross-talk with absorption parameters. At high noise level of 18.0% (Fig. S1 panel (III)), the valley along n becomes quite flat whereas visible curvature persists along m. The contrasts decline further to 67.9% for the n slice and 305.6% for the m slice, indicating that a wide range of n values yield similar costs (poor identifiability), while m remains estimable but with increased uncertaint.

Taken together, these results show that noise preferentially erodes the curvature of the objective along the n dimension, while the amplitude m remains identifiable across realistic noise levels. This observation justifies constraining n to physiologically plausible bounds in out ACO-based fitting informed by the literature with proper boundary limits [29]. Furthermore, in vivo FD-NIRS measurements were compared with published reports to rectify and confirm these constraints, ensuring robustness of the fitting algorithm against noise. Such constraints stabilize the fit, reduce cross-talk with absorption parameters, and preserve the reliability of recovered tissue properties.

### 4.2 Impact of noise amplification in the derivative domain

Simulation results in Fig. 4 demonstrated that relative error increases with noise levels in a noise-sensitive manner for most chromophores. This reflects the inherent sensitivity of derivative-based fitting to spectral noise. Due to the mathematical process of derivation, noise is amplified in the first-derivative stage and further intensified in the second-derivative stage. In other words, noise in the original spectrum, typically appearing as alternating positive and negative fluctuations, is transformed into large spikes with greater variability in the derivative domain. Such enhanced variability diminishes the reliability of chromophore quantification, as genuine absorption features may be obscured by amplified noise artifacts. Nevertheless, the spectrometer employed in our experiments provided a high signal- to-noise ratio (SNR), keeping noise levels below approximately 3–5%. Based on the simulation outcomes, this degree of noise control corresponds to relative errors of no more than 5–10% in the recovered parameters, a level considered acceptable for physiological measurements.

### 4.3 Physiological understanding of in vivo human data

In this study, we quantified absolute chromophore concentrations in vivo using a non-invasive CW-NIRS system. Results from 20 forearms and 18 foreheads (Figs. 6 and 7) showed subject-averaged values were both consistent with prior reports and aligned with established physiological expectations.

The average [HbO] was 25.3 *μ*M in forearms and 34.7 *μ*M in foreheads, while the average [HHb] was 15.3 *μ*M in forearms and 10.5 *μ*M in foreheads. From these values, StO_2_ was calculated as 62.4% in forearms and 76.4% in foreheads. These values fall between the expected physiological limits of arterial (~ 95%) and venous (~ 60%) oxygenation, reflecting the fact that the optical probe samples a bulk tissue volume that includes both arterial and venous vessels. The measuring volume of our probe included a tissue bulk, which allows for measuring averaged oxygenation level from all the arterial and venous vessels. Therefore, we should observe an oxygenation value between general oxygenation limits of arteries and veins. The higher SO_2_ observed in foreheads compared to forearms is also physiologically reasonable. Although the brain constitutes only ~ 2% of total body weight, it consumes ~ 20% of the body’s oxygen to support ATP production required for sustaining life and performing cognitive activities. Consequently, cerebral tissue maintains higher hemoglobin oxygenation compared to peripheral muscle tissue.

Tissue composition results were also consistent with physiology. Water content averaged 80.6% in forearms and 82.3% in foreheads. However, the fat% differed substantially, with 64.3% in forearms compared to 31.8% in foreheads. The brain is often reported as the ‘fattest’ organ in human body, containing ~ 60% fat by dry weight [41]. However, our optical measurements sampled multiple tissue layers, including scalp, skull, white matter, and gray matter. Since fat is concentrated primarily in white matter [42] [42], and is sparse in other layers, the overall measured fraction was lower than the reported value for pure brain tissue (i.e., white and gray matter). In contrast, forearms measurements included the subcutaneous adipose layer and underlying muscle fibers located 0.5–2.0 cm beneath the skin surface. Therefore, the regional fat% in forearm is comparatively higher than the regional readings from forehead.

The [CCO] was measured as 5.7 *μ*M in forearms and 3.4 *μ*M in foreheads, corresponding to 7–14% of the total hemoglobin concentration, a ratio consistent with reported physiological ranges [43]. Moreover, the higher [CCO] observed in human forearms than foreheads also align with known tissue-specific differences. Skeletal muscle contains the highest mitochondrial density among human tissues [44–46]. The flexor digitorum muscle, selected as the forearm measurement site, is a red muscle specialized for repetitive and forceful gripping and lifting. Its high mitochondrial content supports substantial ATP production, leading to elevated levels of [CCO], the terminal enzyme of oxidative phosphorylation [47].

### 4.4 Potential future applications

The absolute quantification algorithm presented in this study holds strong potential for in vivo assessment of diseases linked to abnormal chromophore concentrations. Many pathological conditions, particularly neurodegenerative disorders, are associated with mitochondrial dysfunction and impaired oxygen utilization. The ability to non-invasively quantify absolute [CCO] provides a direct indicator of mitochondrial oxidative metabolism. This parameter could serve as an early biomarker for disease detection and progression monitoring, with potential applications in conditions such as Alzheimer’s disease [13, 19, 48]. Furthermore, the method may enable longitudinal tracking of mitochondrial function in response to therapeutic interventions, thereby supporting both diagnosis and treatment evaluation.

### 4.5 Limitations

Despite the physiologically reasonable results obtained on human forearms and foreheads, several limitations of the current algorithm should be acknowledged. First, the inverse problem is ill-posed and exhibits parameter cross-talk (e.g., [HbO]–[HbO], water–fat, and m-n). Consequently, the recovered solution can be sensitive to the specified bounds. If bounds are too tight, the optimizer may “edge-hit,” biasing estimates toward the boundary; if too wide, the search space expands, increasing variance and susceptibility to local minima. Tissue-, device-, and site-specific physiology (brain vs. muscle; superficial layers vs. deeper cortex) further complicates a single, universal bound set. While we constrained parameters using literature-based ranges, the resulting estimates remain prior-dependent. Future work should test how sensitive the estimates are to different bound choices, and consider using physiological guidance (e.g., Bayesian priors or penalty terms on StO_2_, total hemoglobin concentration, m, and n) to reduce bias from strict cut-offs while keeping results biologically realistic.

Second, relative error rose to ~ 10% when [CCO] < 3 *μ*M, indicating weak identifiability in this regime. This limits sensitivity to small [CCO] changes during mild perturbations such as functional activation or transcranial laser stimulation [24]. Because spectral features of [CCO] partially overlap with hemoglobin and water, the estimator relies heavily on the derivative domain and the bounds/priors for separation. Improving identifiability may require broader wavelength coverage in the red/NIR, refinement of the algorithm, multi-distance measurements, or hierarchical constraints that couple [CCO] to physiologically reasonable ranges of hemoglobin and scattering. Also, while our results are consistent with noninvasive expectations, direct validation against invasive or accepted biochemical/physiological standards remains necessary, especially for [CCO]. Such validation is essential to quantify absolute accuracy beyond relative errors in simulation and controlled experiments.

Finally, potential cross-talk from neglected chromophores in our model may also contribute to relative error. While [HbO], [HHb], [CCO], water, and fat are the dominant absorbers in the NIRS band, minor absorbers such as melanin and collagen can also influence optical signals. To avoid overfitting by including too many parameters, their contributions were assumed negligible, which may introduce extra relative error caused by cross talk of unconsidered chromophores on top of our simulation. However, collagen concentrations in tissue are relatively low and contribute minimally to attenuation, while melanin is primarily confined to the superficial epidermis. Given that our optical probes interrogate depths of ~ 0.7–1.0 cm and ~ 1.7–2.0 cm, melanin’s contribution is expected to be minimal.

## 5. Conclusion

In this study, we demonstrated a non-invasive bb-NIRS method to quantify absolute [CCO] along with other major tissue components and scattering properties. The approach utilized an ant-colony optimization algorithm applied to the first- and second-derivative domains of diffuse reflectance spectra. Simulations with varying chromophore combinations were conducted to evaluate relative accuracy under different noise levels. Subsequently, in vivo measurements from 20 human forearms and 18 human foreheads produced results consistent with concurrent FD-NIRS, prior reports, and established physiological benchmarks. The approach utilized an ant-colony optimization algorithm applied to the first- and second-derivative domains of diffuse reflectance spectra. Simulations with varying chromophore combinations were conducted to evaluate relative accuracy under different noise levels. Subsequently, in vivo measurements from 20 human forearms and 18 human foreheads produced results consistent with concurrent FD-NIRS, prior reports, and established physiological benchmarks.

## Supplementary Material

Supplementary Files

This is a list of supplementary files associated with this preprint. Click to download.

• SupplementalDocument.docx

## Figures and Tables

**Table 1:** Initial parameters used in simulation, including chromophore concentrations ([HbO], [HHb], [CCO]) in *μ*M, tissue composition (water and fat volume fractions), and scattering parameters (***m***: amplitude in cm^−1^; ***n***: scattering power, dimensionless).

Sets	[HbO] (*μ*M)	[HHb] (*μ*M)	[CCO] (*μ*M)	Water%	Fat%	m (cm^−1^)	n
**1**	40	20	5	80%	25%	10	1.2
**2**	30	20	3	80%	25%	10	1.2
**3**	40	20	8	80%	25%	10	1.2
**4**	40	20	3	80%	25%	10	1.2

**Table 2:** Demographic information and reduced scattering properties of participants measured on the human forearm and forehead using FD-NIRS system (F: female; M: male). Reported parameters include subject number, age, sex, ${\mu^{\prime }}_{s}$ at ***λ*** = 750, 785, 811, and 830 nm, and scattering power ***b***. Values are reported separately for forearm and forehead measurements. “N/A” indicates data not available. “Avg.” denotes the group mean, and “Std.” represents the standard deviation.

No.	Age (yr)	Sex	$\mu_{s}^{\prime }$ at *λ* = 750 nm (cm^−1^)		$\mu_{s}^{\prime }$ at *λ* = 785 nm (cm^−1^)		$\mu_{s}^{\prime }$ at *λ* = 811 nm (cm^−1^)		$\mu_{s}^{\prime }$ at *λ* = 830 nm (cm^−1^)		b	
			arm	head	arm	head	arm	head	arm	head	arm	head
**1**	27	M	5.1	11.0	5.0	10.5	4.9	10.1	4.6	9.8	−0.9	−1.1
**2**	28	M	5.5	10.5	5.3	10.0	5.1	9.7	5.1	9.3	−0.8	−1.1
**3**	26	F	4.6	10.6	4.4	10.2	4.3	9.8	4.2	9.6	−0.8	−0.9
**4**	53	F	4.5	N/A	4.3	N/A	4.3	N/A	4.0	N/A	−0.7	N/A
**5**	55	M	5.3	N/A	5.2	N/A	5.0	N/A	4.8	N/A	−0.9	N/A
**6**	43	M	4.8	9.8	4.6	9.5	4.4	9.1	4.3	8.7	−1.1	−1.1
**7**	25	F	4.7	10.4	4.4	10.0	4.3	9.5	4.2	9.0	−1.1	−1.3
**8**	27	M	5.5	9.6	5.3	9.3	5.2	8.8	5.0	8.5	−0.8	−1.2
**9**	37	F	5.6	9.6	5.5	9.5	5.3	9.0	5.2	8.7	−1.2	−0.9
**10**	29	F	5.1	10.1	4.9	9.9	4.7	9.5	4.5	9.0	−1.2	−1.0
**11**	23	F	5.4	9.4	5.1	9.1	5.0	8.7	4.9	8.5	−0.9	−1.0
**12**	25	M	4.6	9.6	4.5	9.3	4.3	9.1	4.2	8.7	−0.9	−0.9
**13**	35	M	6.0	10.0	5.7	9.7	5.6	9.2	5.4	8.8	−0.9	−1.2
**14**	36	F	5.5	10.5	5.2	10.1	5.1	9.7	4.9	9.4	−1.0	−1.0
**15**	37	M	4.9	10.9	4.6	10.5	4.6	10.1	4.5	9.7	−0.7	−1.1
**16**	36	F	5.1	11.1	5.0	10.6	4.8	10.2	4.7	9.9	−0.8	−1.1
**17**	29	F	5.3	10.3	5.1	9.9	5.0	9.5	4.8	9.1	−0.9	−1.1
**18**	36	M	5.0	9.2	4.9	8.9	4.6	8.7	4.5	8.0	−1.0	−1.2
**19**	35	M	4.7	9.7	4.5	9.3	4.4	9.0	4.2	8.6	−1.0	−1.1
**20**	33	M	5.1	11.2	5.0	10.5	4.8	10.2	4.7	9.8	−0.8	−1.2
**Avg.**	33.75	11 M	5.1	10.2	4.9	9.8	4.7	9.4	4.6	9.0	−0.9	−1.1
**Std.**	8.72	9 F	0.4	0.6	0.4	0.5	0.3	0.5	0.3	0.5	0.1	0.1

## Data Availability

Data sets supporting the results presented in this work are not publicly available at this time but may be obtained from the corresponding author upon reasonable request.
